# Traditional Chinese medicine non-pharmaceutical therapies for chronic adult insomnia: A Bayesian network meta-analysis protocol

**DOI:** 10.1097/MD.0000000000017754

**Published:** 2019-11-15

**Authors:** Feizhou Li, Bo Xu, Ping Wang, Ling Liu

**Affiliations:** a Institute of Gerontology; b Clinical College of Traditional Chinese Medicine; c The First Clinical College, Hubei University of Chinese Medicine; d Encephalopathy Department, Hubei Provincial Hospital of Traditional Chinese Medicine, Wuhan, Hubei Province, China.

**Keywords:** Bayesian network meta-analysis, chronic adult insomnia, non-pharmaceutical therapy, protocol, traditional Chinese medicine

## Abstract

**Background:**

Traditional Chinese medicine (TCM) non-pharmaceutical therapies are frequently used for chronic insomnia in China, but in clinical practice, most practitioners choose appropriate treatments based on personal experience. In our study, Bayesian network meta-analysis will be used to identify differences in efficacy and safety between diverse non-pharmaceutical therapies for chronic adult insomnia.

**Methods:**

The authors will totally retrieve seven electronic databases from their establishment to August 2019 in accordance with relevant strategies. After a series of screening, the 2 researchers will employ the Aggregate Data Drug Information System (ADDIS) and R software to analyze the data extracted from enclosed Randomized Controlled Trials (RCTs). Ultimately, the evidentiary grade of the results will be evaluated.

**Results:**

This study will provide reliable evidence for different non-pharmaceutical therapies on chronic insomnia in adults.

**Conclusions:**

The findings will be an available reference to evaluate the efficacy and safety of different non-pharmaceutical therapies on chronic insomnia in adults and may provide decision-making reference on which method to choose for clinicians.

**Trial registration number:**

PROSPERO CRD42019141496.

## 1 Introduction

Insomnia refers to a subjective experience of difficulty in falling asleep, difficulty in maintaining sleep and early awakening after excluding factors such as environment and insufficient sleep opportunities, usually accompanied by impairment of daily functions during the day.^[1]^ Insomnia is the most common sleep disorder in adults with a pooled prevalence of 15% in China.^[2]^ Furthermore, the incidence in the special population is higher than that in the general population. A systematic review concluded that the weighted mean incidence of insomnia among university students was 18.5%,^[3]^ which was higher than that in the normal population. One Chinese cross-sectional study^[4]^ of the elderly in Anhui Province showed that 24% of the elderly suffered from insomnia. Not only do we need to pay attention to the high incidence, but the threat to human health cannot be underestimated. The latest researches discovered that insomnia could increase the risk of cardiovascular disease, hypertension, chronic kidney disease, and depression.^[5–8]^

At present, therapies for insomnia could be generally divided into psychotherapy and pharmacotherapy. Cognitive behavioral therapy for insomnia (CBT-I), as a main kind of psychotherapy, has been recommended as the first-line option for chronic adult insomnia by the European insomnia guideline.^[9]^ Nevertheless, apparently insufficient professional therapists and poor compliances are important problems and obstacles for insomniacs to obtain CBT-I.^[10,11]^ Moreover, CBT-I cannot be widely used in most clinical settings especially in developing countries like China,^[12]^ which also limited its clinical practice. Currently, medications recommended for chronic adult insomnia in China mainly include benzodiazepines, non-benzodiazepine hypnotics, melatonin receptor agonists, orexin receptor antagonist, and the antidepressant drugs with hypnotic effects.^[13]^ Whereas, adverse reactions are vital factors restricting the clinical application of these drugs.^[14]^

In light of the limitations of the above treatments, TCM, as an essential component of complementary and alternative medicine, has gained more and more attention for insomniacs. As we all know, TCM is famous for its clinical application of herbal medicine and acupuncture, but actually, there are many other non-pharmaceutical treatments in TCM, which have also been widely used by TCM practitioners for a long time in medical practice, such as TCM Psychotherapy, aromatherapy, musicotherapy, moxibustion, tuina, scraping, and TCM exercise therapy like Tai Chi Chih, Qigong, Wuqinxi, Baduanjin.

Non-pharmacological interventions like acupuncture (involving penetrating thin, solid, stainless steel needles into specific points that are operated by hand or electrical stimulation),^[15]^ moxibustion (stimulating specific areas of human skin by igniting moxa),^[16]^ tuina (Chinese massage, a wide range of technical operations performed by a doctor's fingers, hands, elbows, knees, or feet on the muscles or soft tissues at specific body locations),^[17]^ and scraping (using a smooth scraping board with some massage oil to repeat the scrape in sequence at specific body locations)^[18]^ have been developed and employed in the management of insomnia. TCM psychotherapy generally refers to conventional TCM psychotherapy and modern TCM psychotherapy.^[19]^ Low resistance state thought induction psychotherapy based on TCM theory is an important method of modern TCM psychotherapy for insomnia. Through integrating TCM Qigong, psychological hypnotic and so on, the patient is induced to the low resistance state, in which the patient can accept the information without resistance, so as to carry on the idea introduction treatment at the subconscious level.^[20]^ The psychological treatment idea of using emotion to solve psychological illness like insomnia, based on the holistic concept and the five elements theory, is the main means of conventional TCM psychotherapy.^[19]^ Aromatherapy, as a substitute or adjunctive treatment, has unique effects in promoting health and treating certain diseases like insomnia.^[21,22]^ Musicotherapy can improve the quality of sleep, shorten the latency of sleep onset, improve sleep efficiency, and has the advantages of non-invasive, cheap and no adverse reactions.^[23]^ In China, researchers usually apply some elements of traditional Chinese culture in their studies, for example, Guqin.^[24]^ In addition, the published evidence-based medical documents also confirm that TCM exercises such as Tai Chi Chih and Baduanjin have potentially positive effects on insomnia management.^[25,26]^

Although above systematic reviews with or without meta-analysis have analyzed the effectiveness of different TCM therapies for insomnia, no comparison of efficacy between different non-pharmacological therapies has been made. As a result, there is no decision-making conclusion as to which method to choose in clinical practice. Therefore, the authors aim to examine the comparative effectiveness of TCM non-pharmacological therapies for chronic adult insomnia by conducting a Bayesian network meta-analysis.

## 2 Methods

### 2.1 Protocol and registration

The authors have formulated this protocol according to the commonly accepted standards, namely Preferred Reporting Items for Systematic Reviews and Meta-Analyses Protocols (PRISMA-P).^[27]^ The authors have obtained the registration number (CRD42019141496) of this study on PROSPERO platform (https://www.crd.york.ac.uk/PROSPERO/).

### 2.2 Ethics

Given that the meta-analysis will not involve the collection of privacy information, ethical approval is not necessary for our research.

### 2.3 Eligibility criteria

The PICOS (participants, interventions, comparisons, outcomes, and study design) principle are the 5 main factors determining the inclusion and exclusion criteria of this study.

#### 2.3.1 Type of participants

All adults (greater than or equal to 18 years old) clearly diagnosed with chronic insomnia will be included. The diagnostic criteria used can be one of the following: Diagnostic and Statistical Manual of Mental Disorders (DSM),^[28]^ International Classification of Sleeping Disorders (ICSD),^[29]^ International Classification of Diseases (ICD),^[30]^ Chinese Classification and Diagnosis of Mental Disorders (CCMD),^[31]^ and Guidelines for the Diagnosis and Treatment of Adult Insomnia in Chinese.^[13,32]^

#### 2.3.2 Type of interventions and comparators

TCM non-pharmaceutical therapies for treating chronic adult insomnia include TCM Psychotherapy, aromatherapy, exercise therapy (such as Tai Chi Chih, Qigong, Wuqinxi, Baduanjin, etc), music therapy, acupuncture, moxibustion, tuina, and scraping. These interventions can be used alone or in combination. However, acupoint injection, acupoint application, medicated bath and other treatments containing Chinese herbal medications will be eliminated. Controlled interventions included placebo, basic treatment, or other positive interventions.

#### 2.3.3 Type of outcomes

*Primary outcomes*: The primary outcomes include objective sleep parameters and the Pittsburgh sleep quality index (PSQI).^[33]^ The objective sleep parameters include but not limit to Sleep Latency, Total Sleep Time, Wake After Sleep Onset, and Sleep Efficiency, which can be acquired by polysomnography, actigraphy, electroencephalography and so forth.

Secondary outcomes:

1. Subjective sleep parameters extracted from sleep diaries, for example, Pittsburgh Sleep Diary.^[34]^
2. Sleep score measured by other standardized scales related to sleep, for example, Insomnia Severity Index.^[35]^
3. Daytime function measured by standardized sleep-related scales, for example, the Epworth Sleepiness Scale.^[36]^
4. Quality of life obtained from the corresponding scale.
5. Adverse events and economic costs may also be taken into consideration.

#### 2.3.4 Study design

We will recruit parallel designed RCTs, whether or not the blind method is adopted and regardless of population characteristics, publication status, and duration of trials. However, only Chinese and English literature will be made available for this study. Additionally, the authors will remove quasi-RCTs, duplications, animal trails, review documents, clinical experience, and case reports.

### 2.4 Database and search strategy

The 7 databases, which will be electronically searched for RCTs from inception of each database to August 2019, are as follow: China National Knowledge Infrastructure (CNKI), China Biological Medicine Database (CBM), Chinese Scientific Journals Database (VIP), Wanfang Database, PubMed, EMBASE Database, and Cochrane Central Register of Controlled Trials (CENTRAL). The strategy will be confined to English or Chinese literature. The MeSH terms along with free words will be employed to literature retrieval. The preliminary retrieval strategy for PubMed is provided in Table 1, which will be adjusted in accordance with specific databases. Besides, based on snowball strategy, reference documents from inclusive literature and previous reviews in this field will also be manually checked for other possibly relevant articles.

**Table 1:**
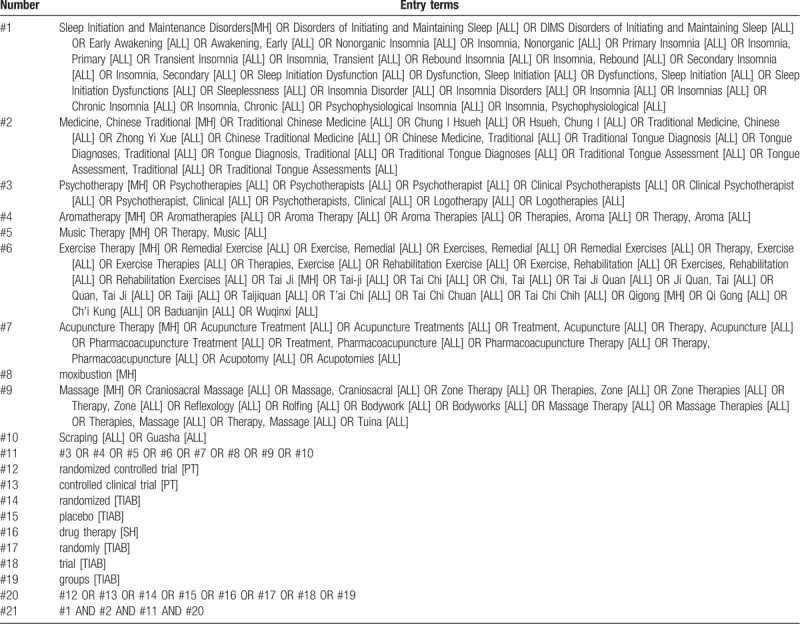
PubMed search strategy draft.

### 2.5 Studies screening

**Figure 1:**
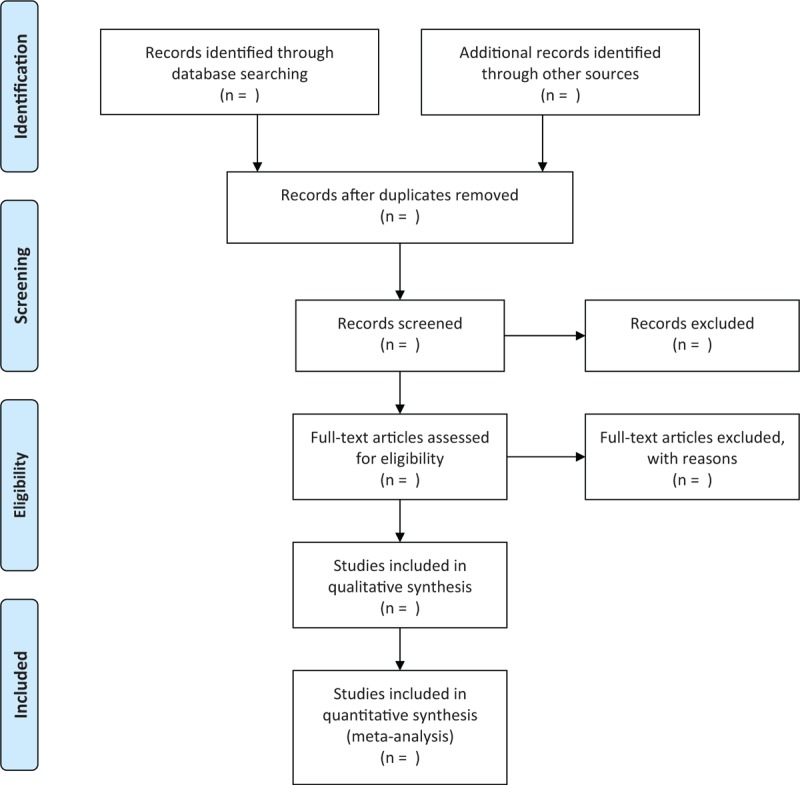
Flow chart of study selection.

The 2 researchers will retrieve the network database mentioned above according to the above retrieval strategies. All qualified documents will be extracted in the form of title and abstract, and preliminary screening will be conducted based on this information. On the basis of the previous step, the full text of the qualified literature will be obtained and further screened. All screening processes will be performed independently by the 2 authors, and the reasons for each rejection will be documented. A third reviewer will be invited to make a final decision on the divergent literature. The flow chart of literature screening is presented in Figure 1.

### 2.6 Data extraction

After screening the literature, the two authors will independently extract the information contained in the eligible literature to form a document feature table, which may include the first author, country, year, randomization procedure, diagnostic criteria, number of centers, sample size, mean age, intervention and comparator, dose, course of treatment, outcome measures, and follow-up time.

### 2.7 Study quality evaluation

For all the inclusive literature, the authors will make a corresponding assessment of its bias risk. The assessment tool is provided by Cochrane, which includes seven items: random sequence generation, assignment concealment, blindness to researchers and subjects, blind evaluation of study outcome, integrity of outcome data, selective reporting of research results and other sources of bias. Each item will be evaluated at three levels: low risk, unclear, and high risk. The above steps will be completed by two authors independently, and a third author will be invited to assist in the decision if necessary.

### 2.8 Data synthesis and statistical methods

#### 2.8.1 Pairwise and network meta-analysis

First of all, the authors will conduct a conventional pairwise meta-analysis of the direct comparison results obtained from the literature. Secondly, for the results of indirect comparison, the authors will employ ADDIS^[37]^ and R software to conduct network meta-analysis based on random effect model. Next, we will calculate the pooled estimates and 95% confidence interval of the mean difference and odds ratios for primary outcomes only. To present indirect comparisons of non-pharmaceutical therapies, we will make a network diagram. The network graph is mainly composed of nodes and lines. Among them, the node represents a kind of therapy, and the nodes connected by lines indicate that there is a direct or indirect comparative relationship between the 2. The node size represents the number of subjects receiving this therapy. The thickness of the line represents the number of studies. Then, we will analyze the outcomes from all direct or indirect comparisons to assess which non-pharmaceutical therapy for adult chronic insomnia is most effective and estimate the rank probabilities of all the groups based on the Markov chain Monte Carlo method.

#### 2.8.2 Assessment of heterogeneity

Clinical and methodological heterogeneity will be evaluated by closely checking the features of the population, treatments and outcomes of the inclusive studies and comparing fit of the fixed effect model and random effect model. For each paired comparison, statistical heterogeneity would be evaluated by the *I*^2^ index. Substantial heterogeneity will be considered where *I*^2^ is >50%.

#### 2.8.3 Subgroup and sensitivity analyses

If considerable heterogeneity is found, subgroup analysis would be envisaged to perform to investigate probable sources of heterogeneity in accordance with the duration of treatment, age, history of insomnia, and research quality. In order to ascertain the sensitiveness of results to modifications in initial assumptions, the authors will conduct sensitivity analysis by only including studies with low risk of bias.

#### 2.8.4 Assessment of inconsistency

Inconsistency means divergences between different sources of evidence. Once a loop is established among interventions, the authors will assess the inconsistency among both direct and indirect evidence. The node-split method will be utilized to determine the location of the inconsistency. Whenever necessary, the authors will seek to detect inconsistencies from all potential network components using the design-by-treatment interaction model and the *I*^2^ index.

#### 2.8.5 Publication bias

Only if each subgroup covers an adequate amount of research (no less than 10 trials) would a funnel plot be used to evaluate publication bias visually.

### 2.9 GRADE quality assessment

Consulting GRADE handbook,^[38]^ the assessment which would be carried out through the Grading of Recommendations Assessment, Development, and Evaluation (GRADE, https://gradepro.org/) by 2 independent authors will be designated into four grades: high quality, moderate quality, low quality, and very low quality.

## 3 Discussion

In spite of a growing number of studies on TCM non-pharmacological treatments for adult patients with chronic insomnia in the late years, there is rare evidence to validate the difference in efficacy and safety among various non-drug therapies. Considering that high-quality meta-analysis could provide reliable guidance for clinicians, the authors intend to complete a network meta-analysis based on Bayesian model. This is the original intention of the draft protocol. Through direct or indirect comparison, the author plans to rank the efficacy and safety of the above 8 non-drug therapies. To our knowledge, it will be the first attempt to perform a Bayesian meta-analysis which compares 8 TCM non-pharmacological therapies for chronic adult insomnia. We hope that the study results will help to figure out which one or which combination of these interventions has the relatively optimal effect and safety and provide decision-making reference for clinicians, patients, and policy-makers to a certain extent.

There are some limitations that may affect the drawn conclusion in this study protocol. There are several methods for TCM exercise therapy as mentioned above. However, the methods of Tai Chi Chih, Baduanjin, Wuqinxi, and Qigong are considered to be the same therapy in this protocol, which may cause excessive heterogeneity. In addition, due to the limitations of language that researchers mastered, the authors expect to look up only English and Chinese literature, which may lead to the potential risk of omitting essential literature.

## Author Contributions

**Conceptualization:** Ping Wang and Feizhou Li.

**Data curation:** Feizhou Li and Bo Xu.

**Formal analysis:** Feizhou Li and Bo Xu.

**Methodology:** Bo Xu and Feizhou Li.

**Software:** Feizhou Li and Bo Xu.

**Supervision:** Ping Wang.

**Writing – original draft:** Feizhou Li and Bo Xu.

**Writing – review & editing:** Ping Wang and Ling Liu.

Ping Wang orcid: 0000-0001-8049-2495

Feizhou Li orcid: 0000-0002-9465-1001

Bo Xu orcid: 0000-0001-9262-6059
